# Supplementary figures and images for: Role of BRCA2 DNA-binding and C-terminal domain in its mobility and conformation in DNA repair
Source: eLife. 2021 Jul 13;10:e67926. doi: 10.7554/eLife.67926 (PMC8324294; doi:10.7554/eLife.67926)

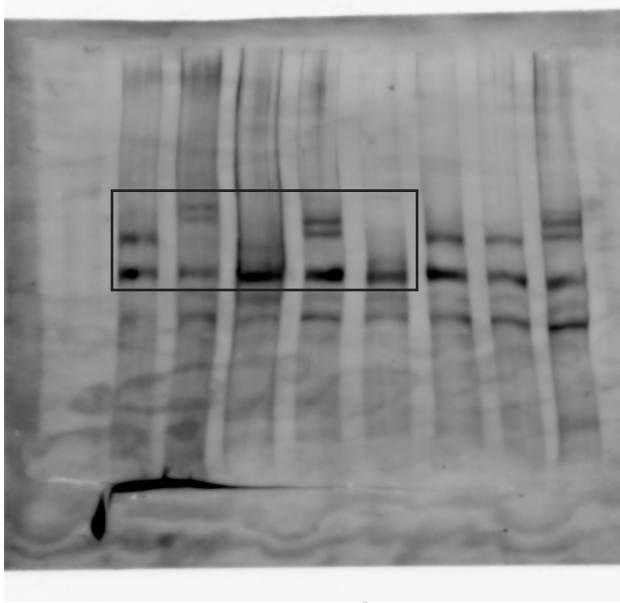

$\alpha$ -mBRCA2

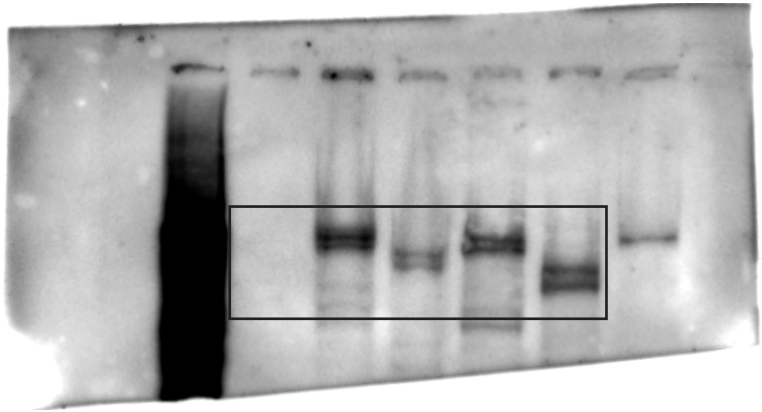

$\alpha$ -HaloTag

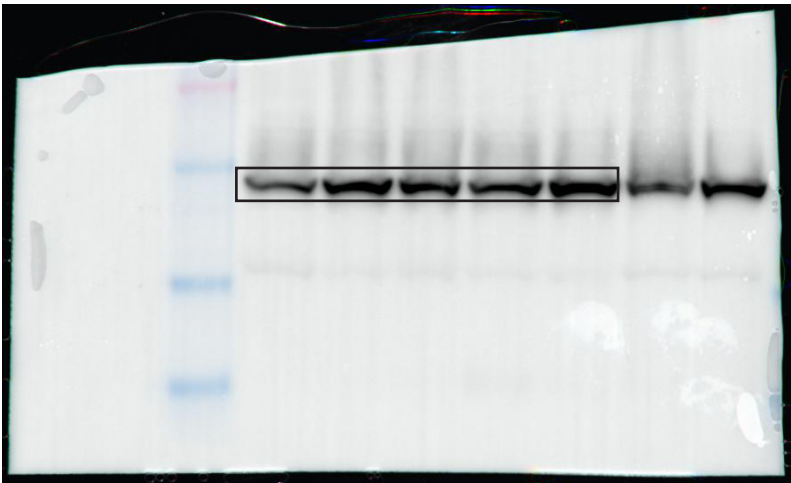

$\alpha$ -PARP-1

Supplement: Figure 1—source data 1. [file elife-67926-fig1-data1.zip › Figure 1 - source data file 1.pdf]

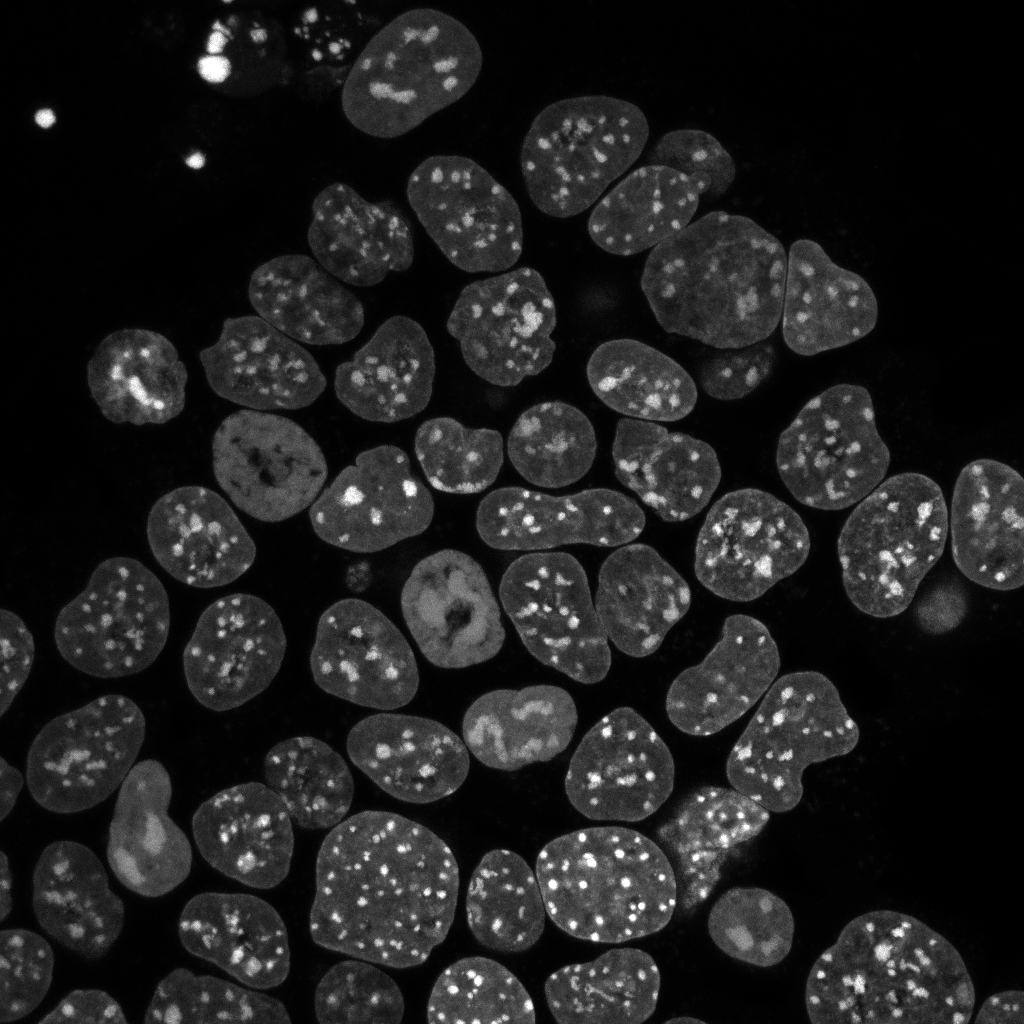

Supplement: Figure 2—source data 2. — Confocal z-projection images. [file elife-67926-fig2-data2.zip › 001 dBDB_IR.tif]

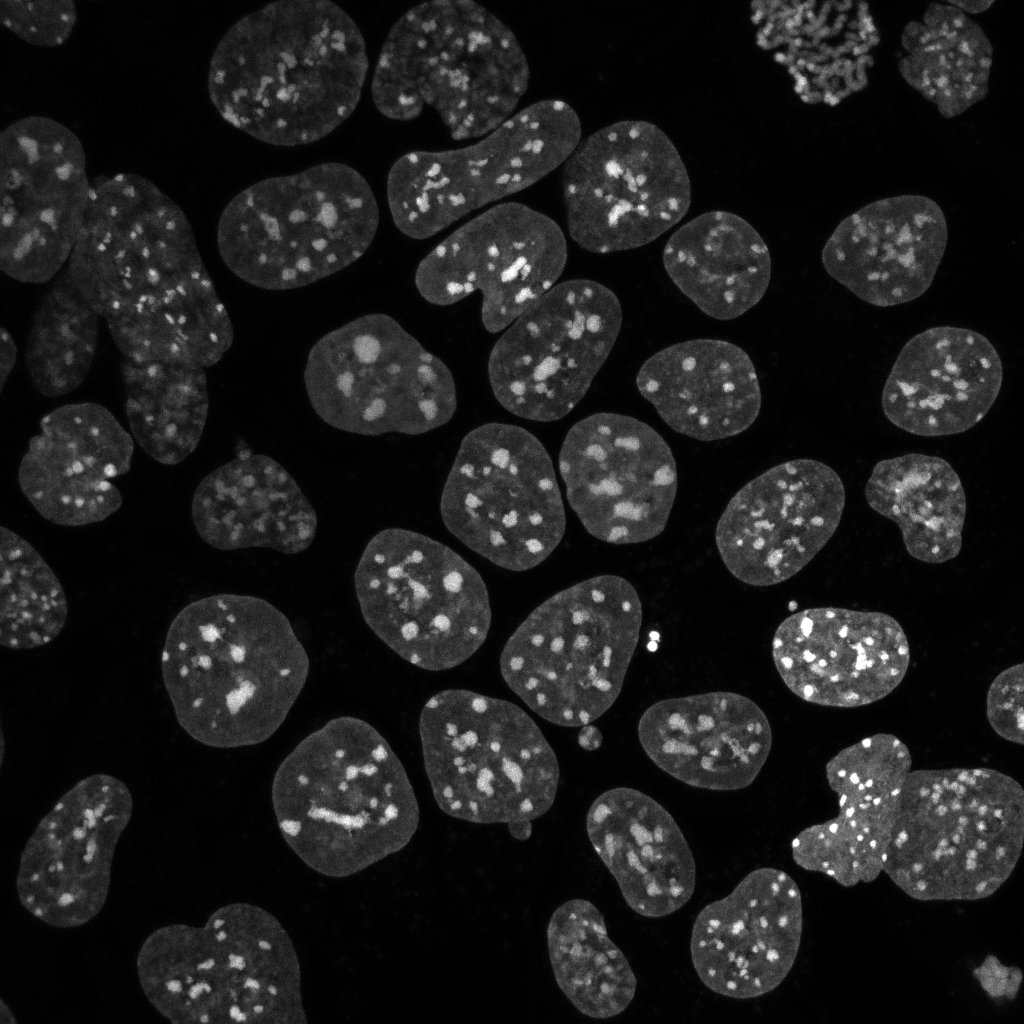

Supplement: Figure 2—source data 2. — Confocal z-projection images. [file elife-67926-fig2-data2.zip › 001 dBDB_noIR.tif]

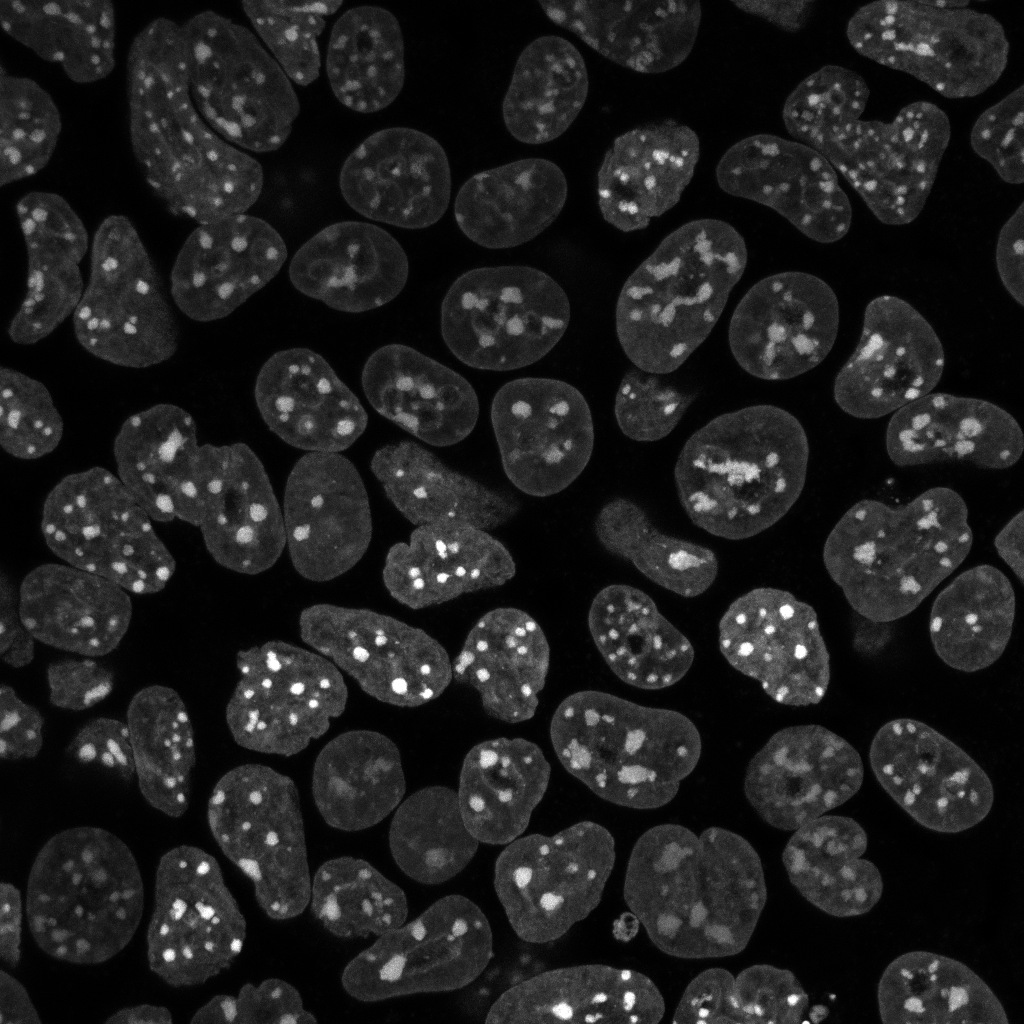

Supplement: Figure 2—source data 2. — Confocal z-projection images. [file elife-67926-fig2-data2.zip › 001 dCTD_IR.tif]

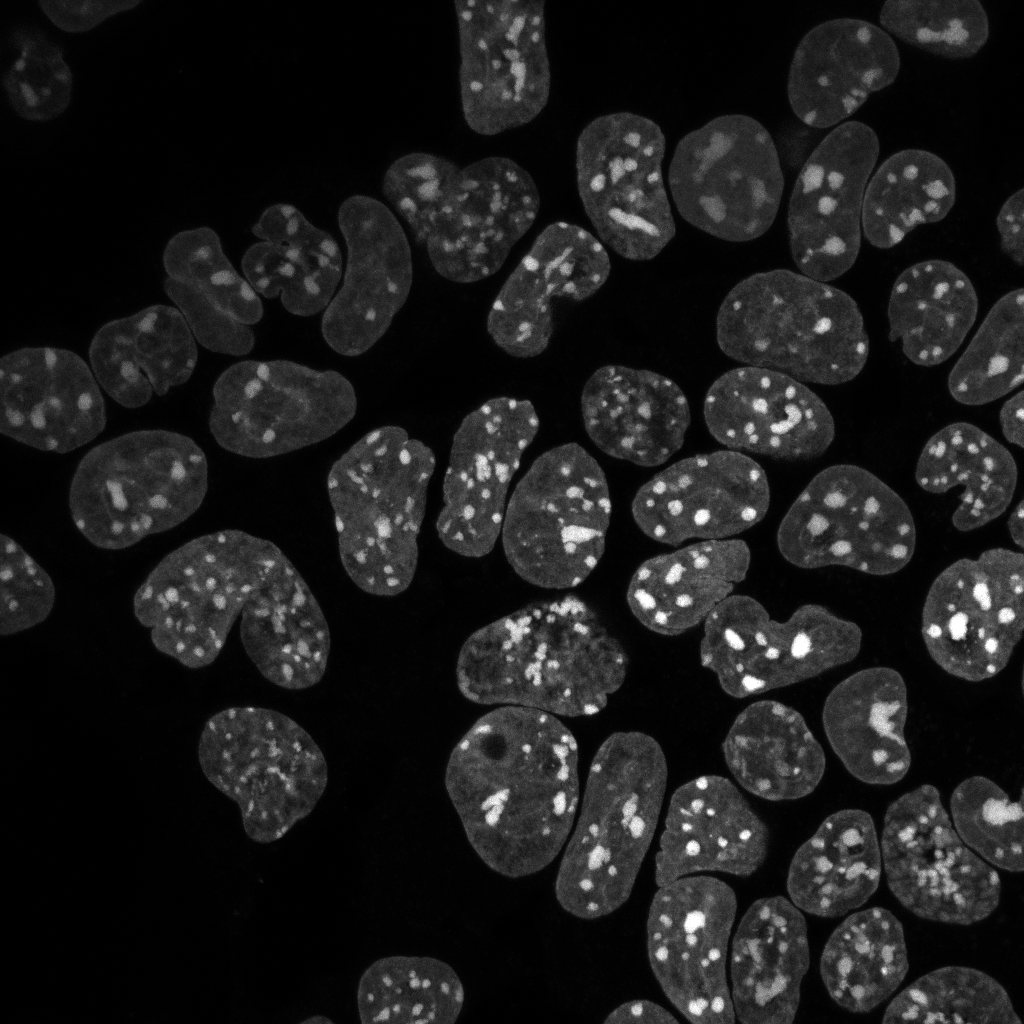

Supplement: Figure 2—source data 2. — Confocal z-projection images. [file elife-67926-fig2-data2.zip › 001 dCTD_noIR.tif]

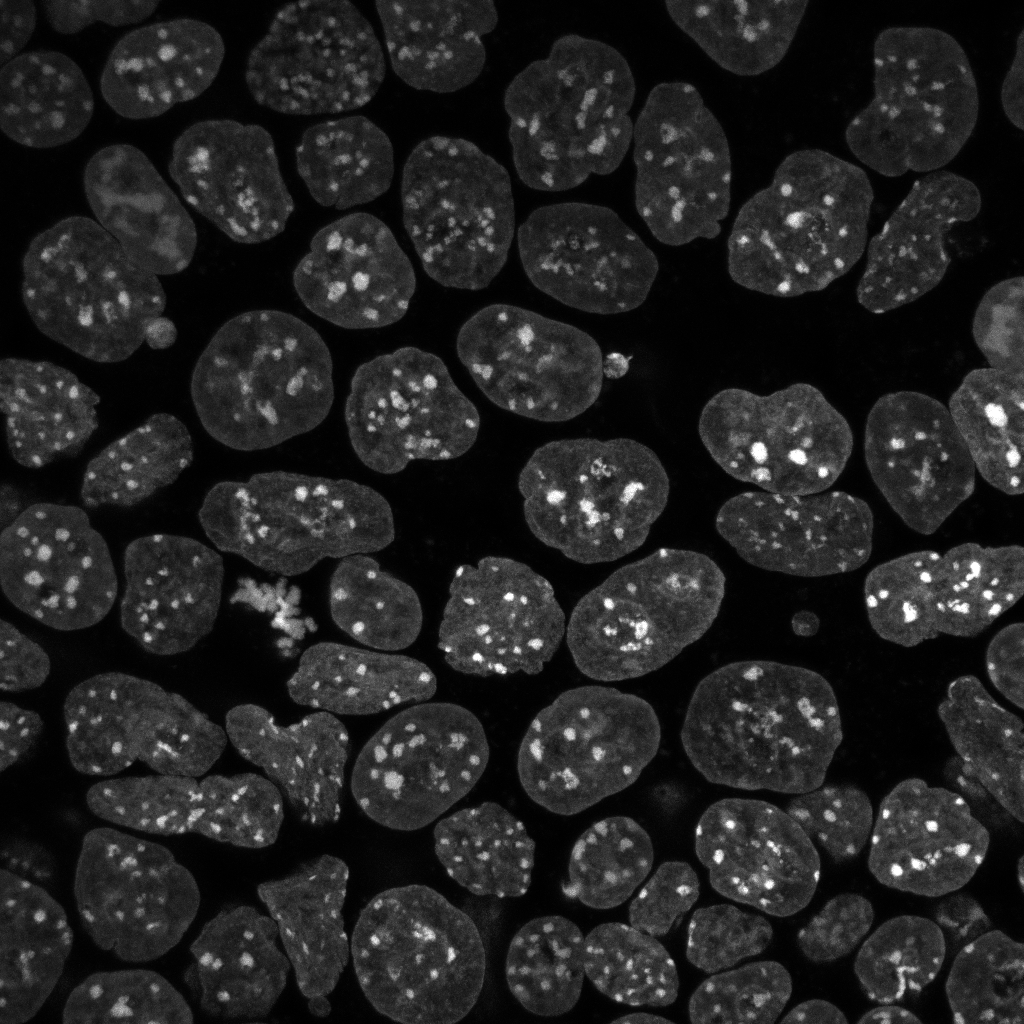

Supplement: Figure 2—source data 2. — Confocal z-projection images. [file elife-67926-fig2-data2.zip › 001 dDBDdCTD_IR.tif]

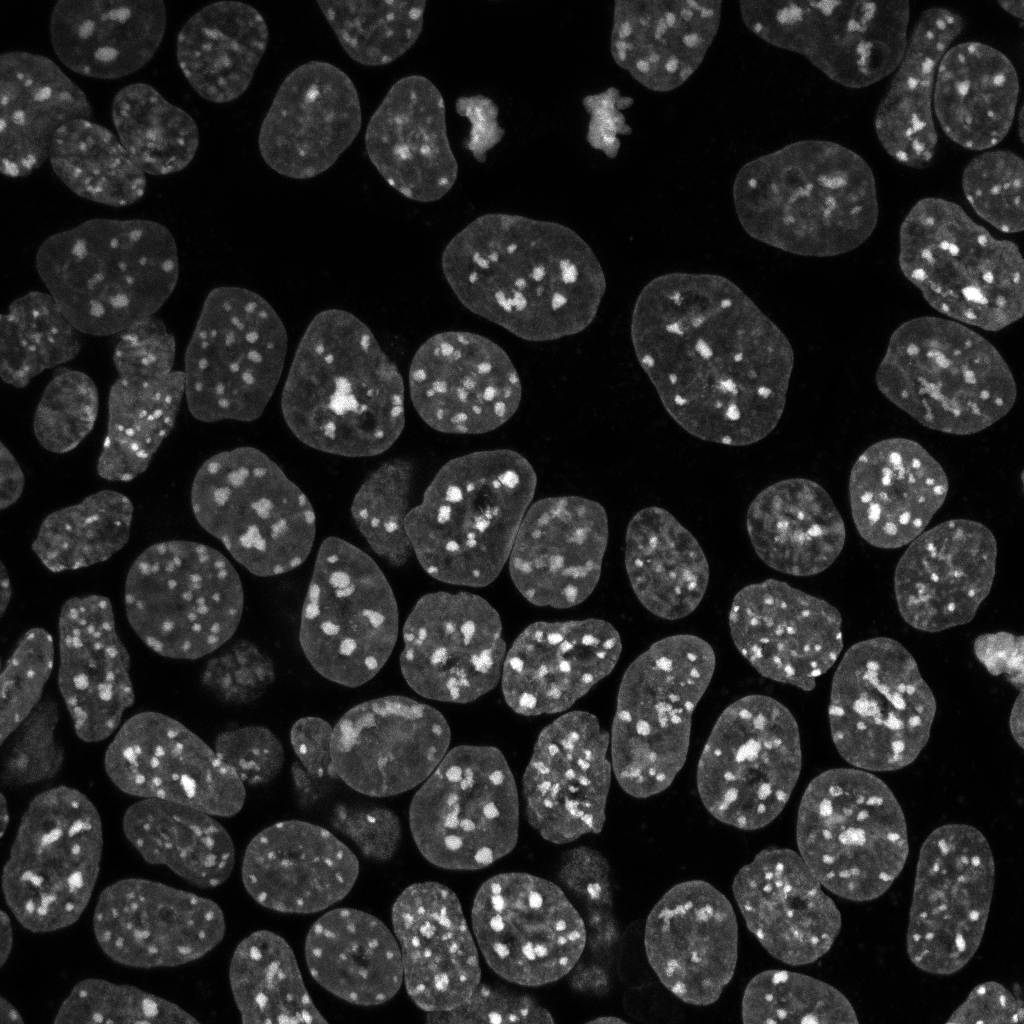

Supplement: Figure 2—source data 2. — Confocal z-projection images. [file elife-67926-fig2-data2.zip › 001 dDBDdCTD_noIR.tif]

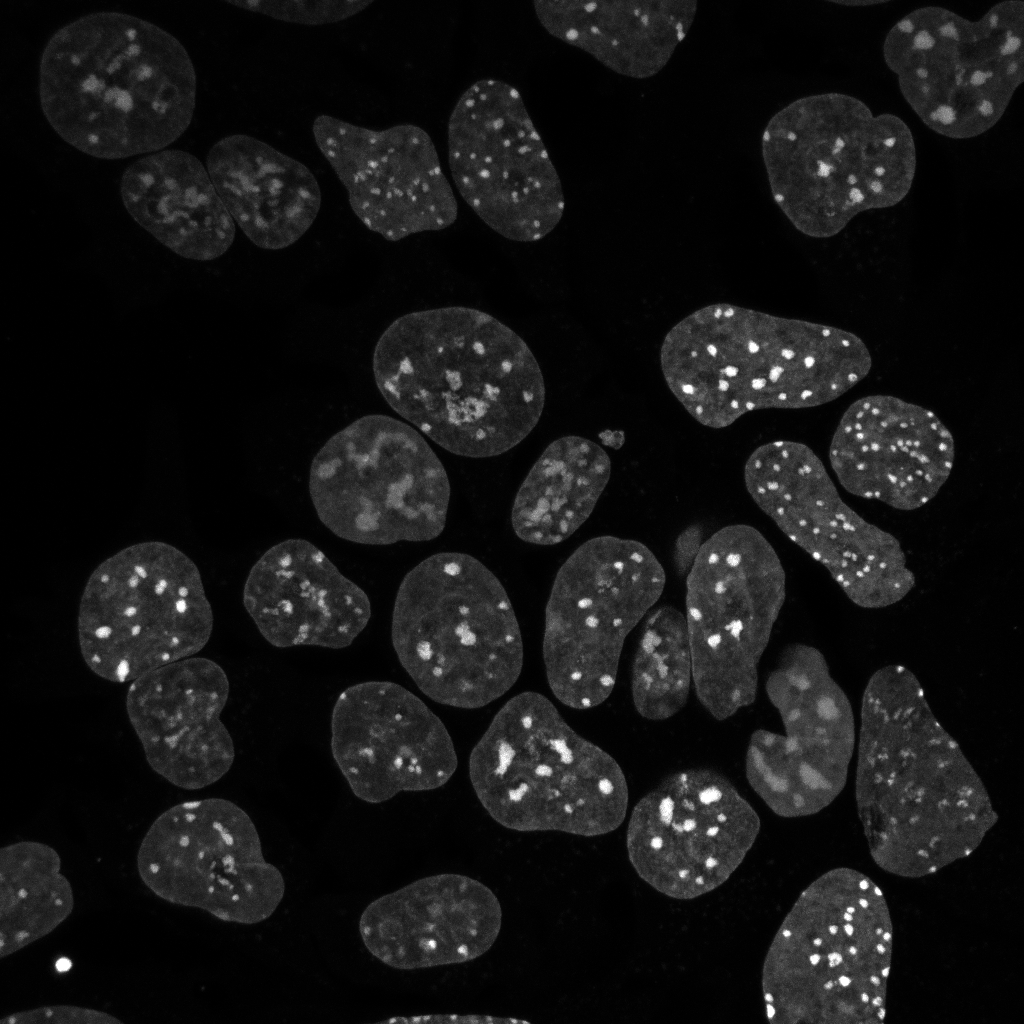

Supplement: Figure 2—source data 2. — Confocal z-projection images. [file elife-67926-fig2-data2.zip › 001 WT_IR.tif]

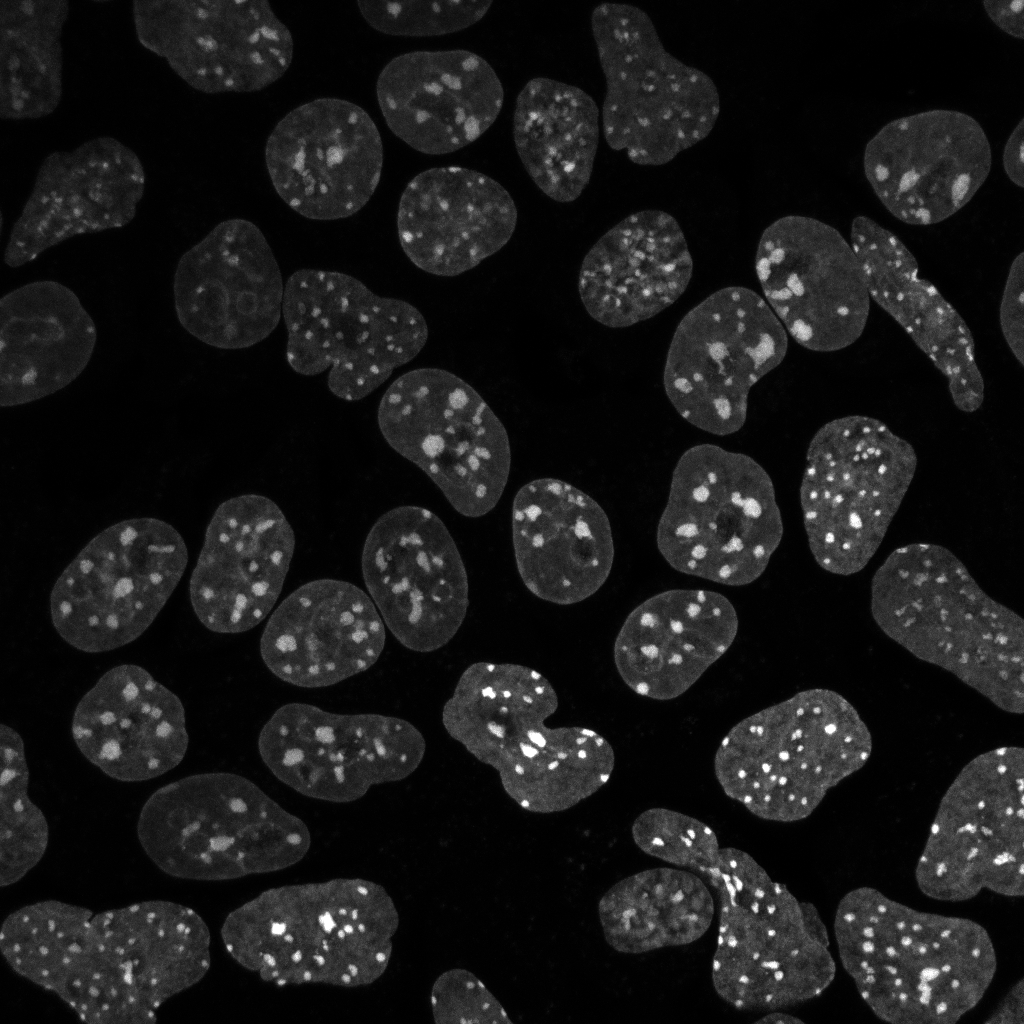

Supplement: Figure 2—source data 2. — Confocal z-projection images. [file elife-67926-fig2-data2.zip › 001 WT_noIR.tif]

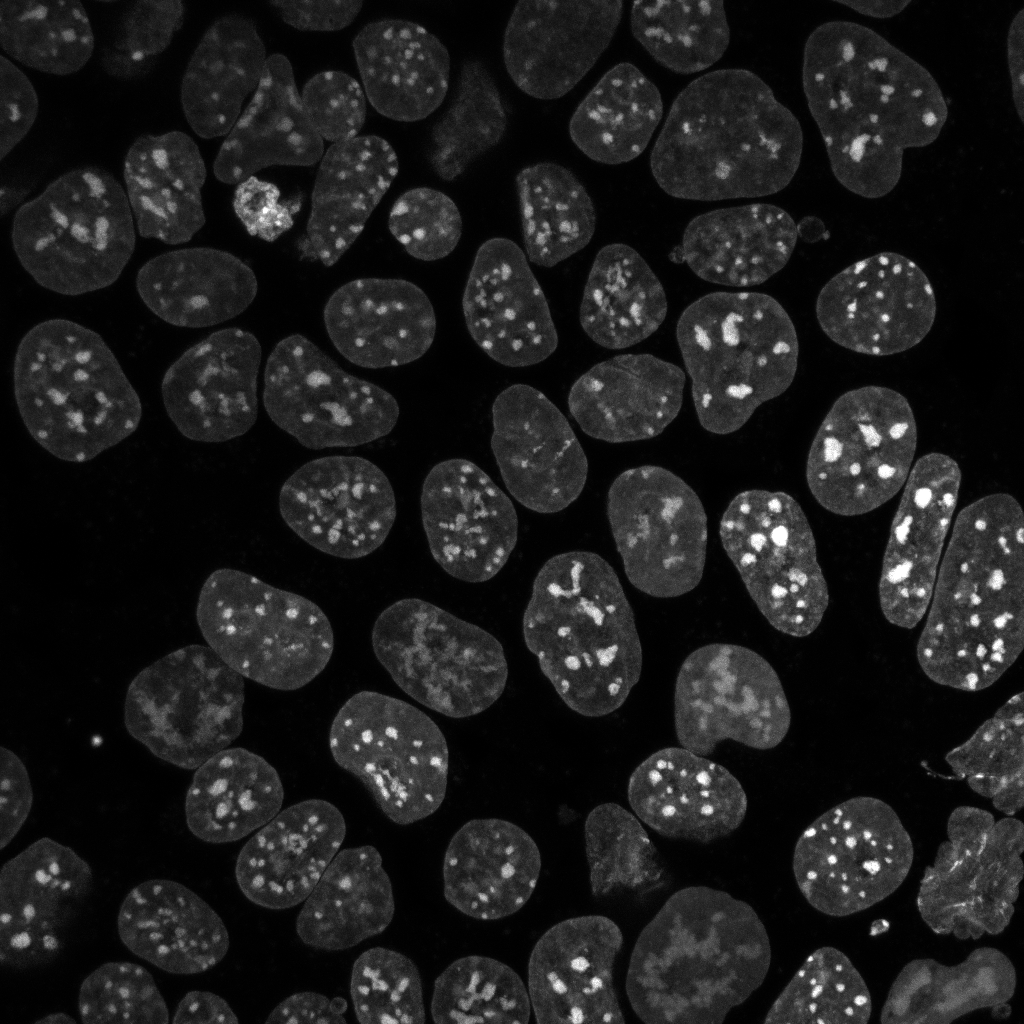

Supplement: Figure 2—source data 2. — Confocal z-projection images. [file elife-67926-fig2-data2.zip › 002 dBDB_IR.tif]

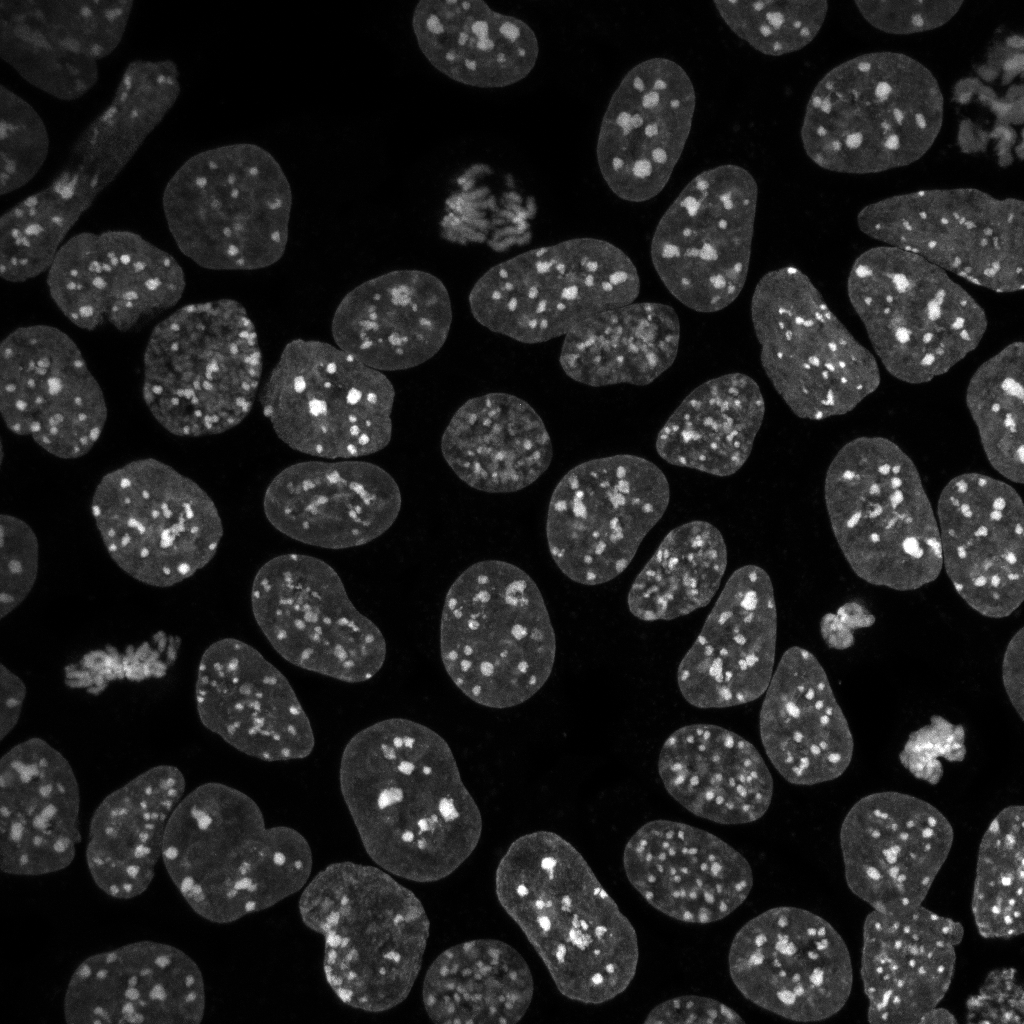

Supplement: Figure 2—source data 2. — Confocal z-projection images. [file elife-67926-fig2-data2.zip › 002 dBDB_noIR.tif]

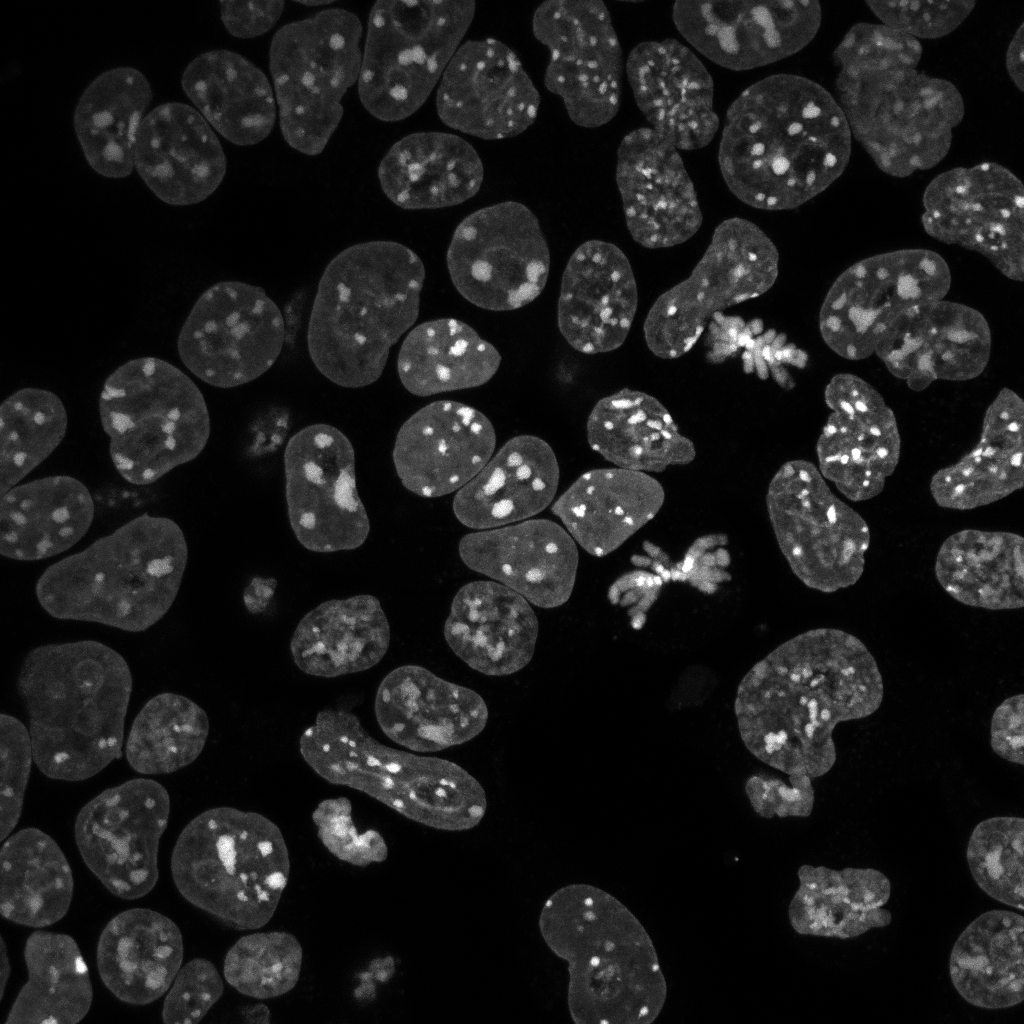

Supplement: Figure 2—source data 2. — Confocal z-projection images. [file elife-67926-fig2-data2.zip › 002 dCTD_noIR.tif]

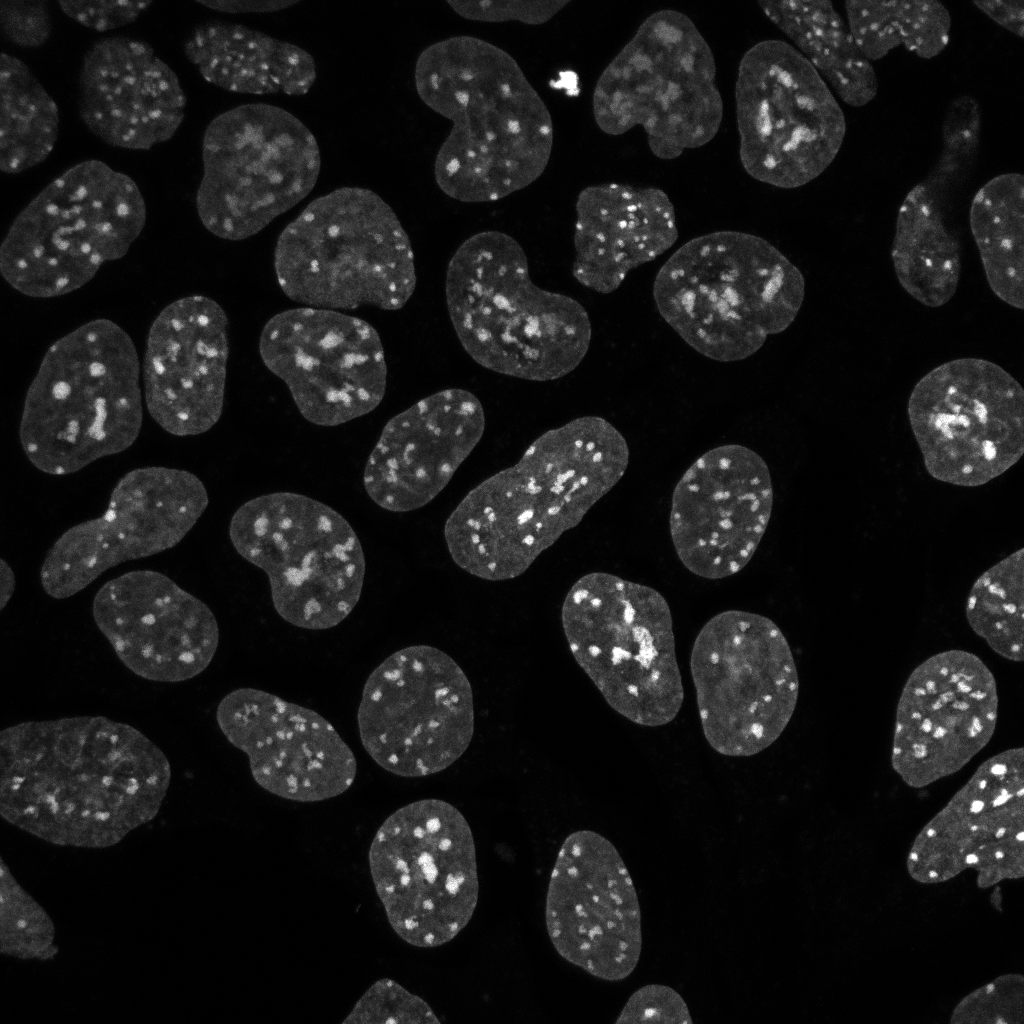

Supplement: Figure 2—source data 2. — Confocal z-projection images. [file elife-67926-fig2-data2.zip › 002 dDBDdCTD_IR.tif]

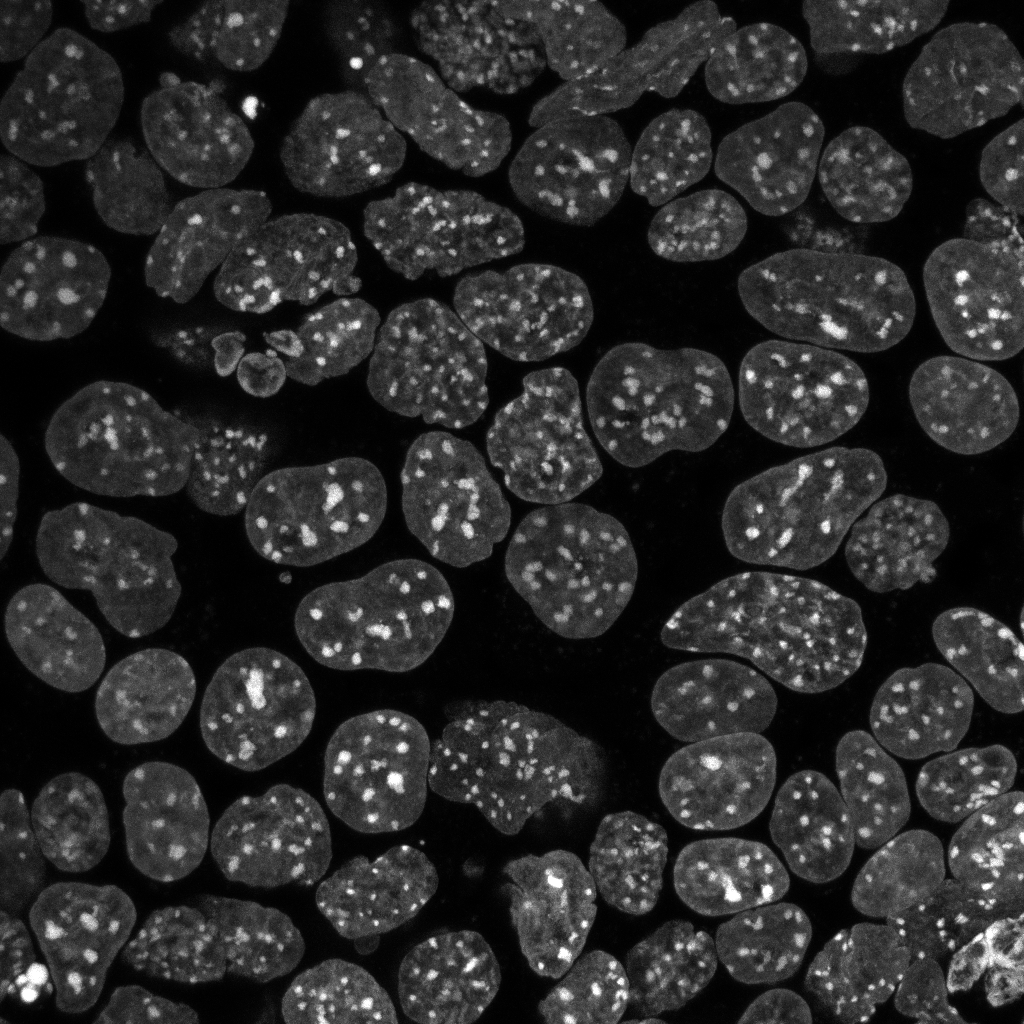

Supplement: Figure 2—source data 2. — Confocal z-projection images. [file elife-67926-fig2-data2.zip › 002 dDBDdCTD_noIR.tif]

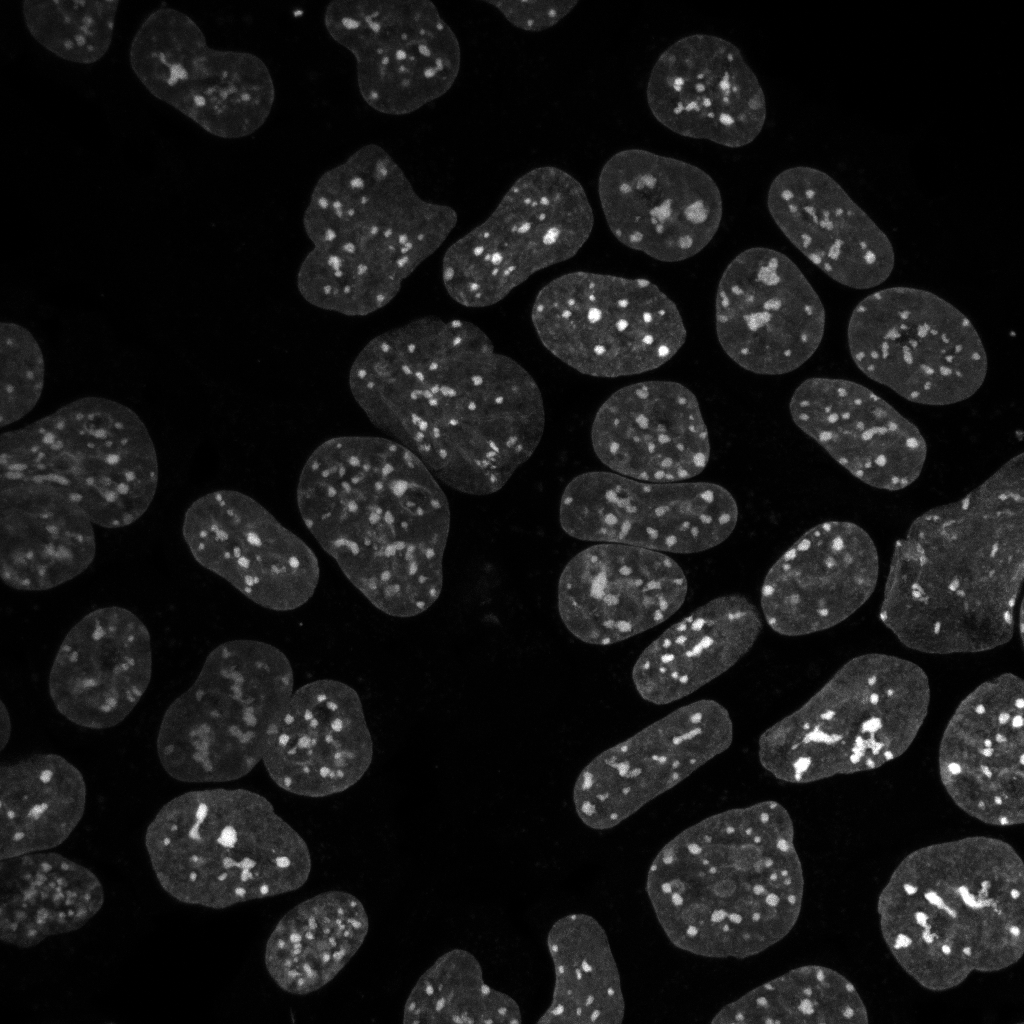

Supplement: Figure 2—source data 2. — Confocal z-projection images. [file elife-67926-fig2-data2.zip › 002 WT_IR.tif]

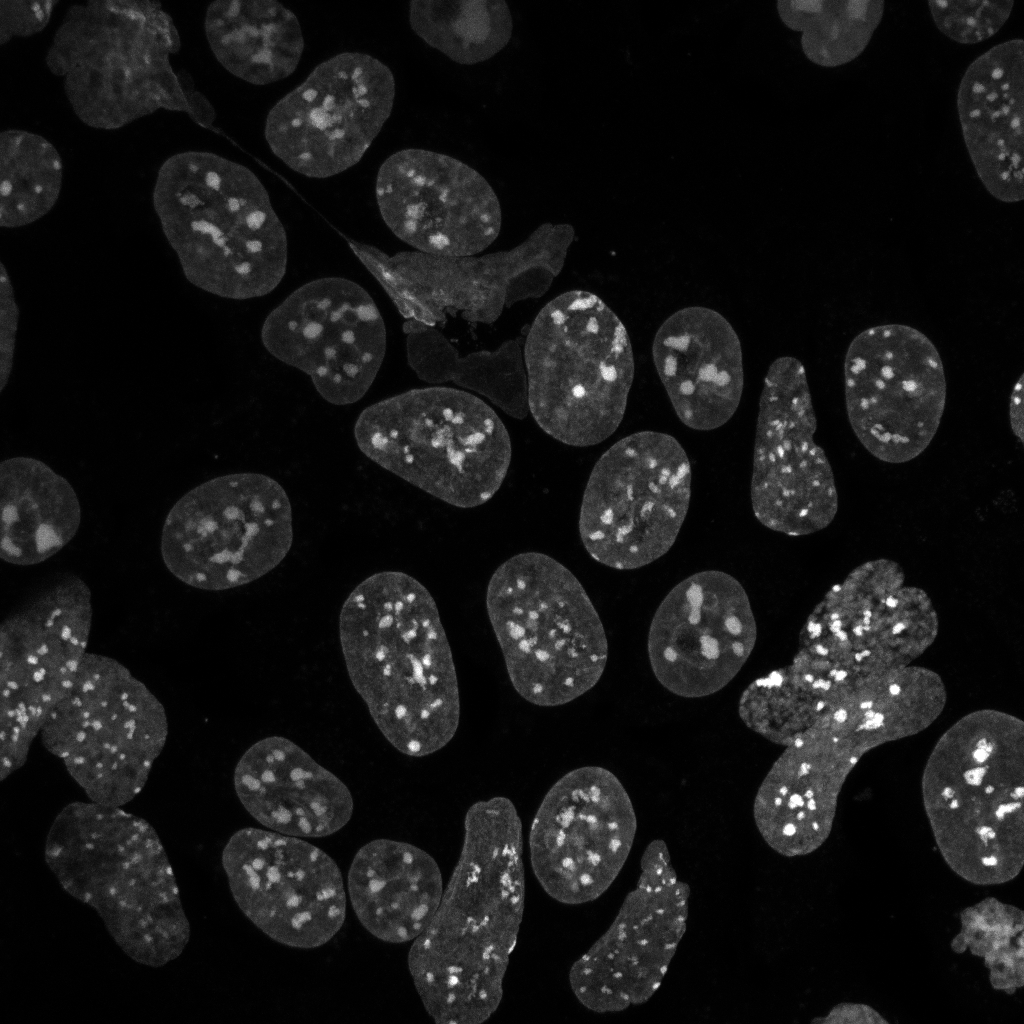

Supplement: Figure 2—source data 2. — Confocal z-projection images. [file elife-67926-fig2-data2.zip › 002 WT_noIR.tif]

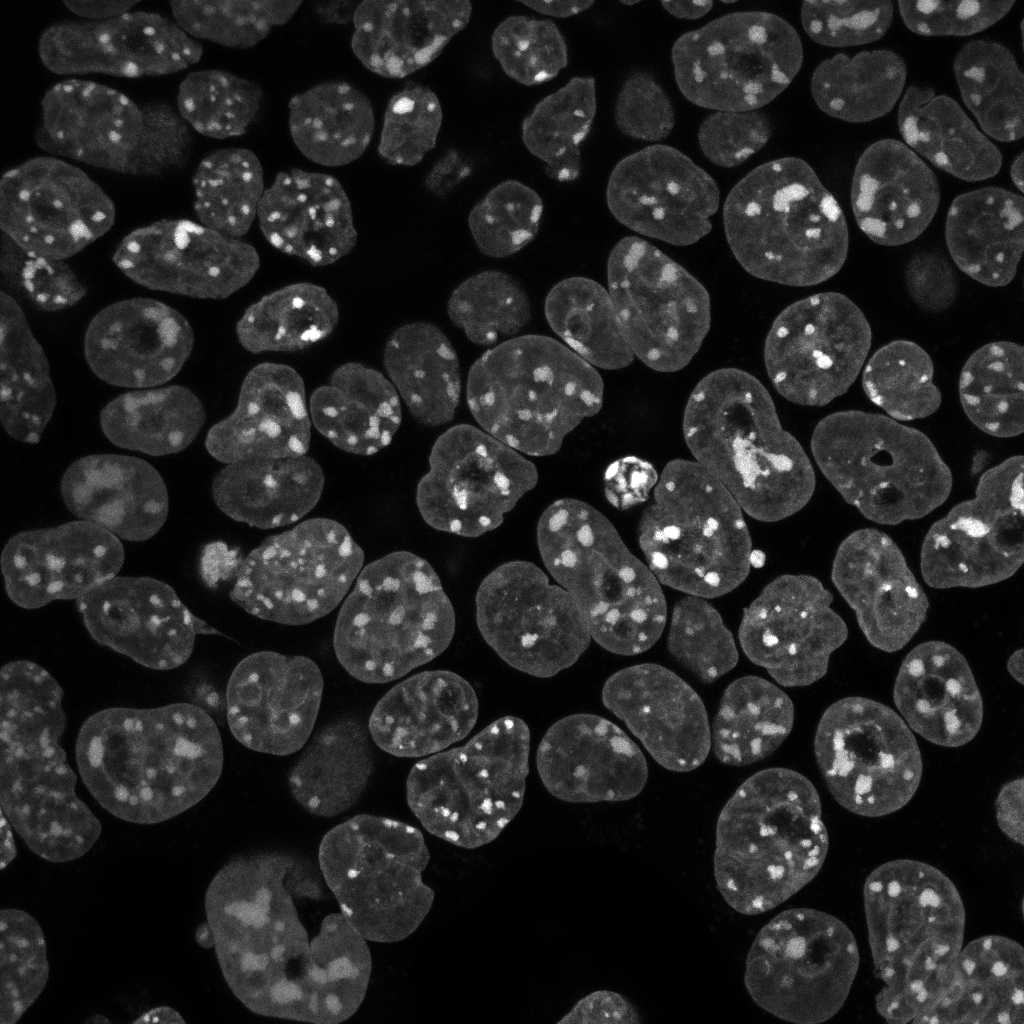

Supplement: Figure 2—source data 2. — Confocal z-projection images. [file elife-67926-fig2-data2.zip › 002 dCTD_IR.tif]
